# Genome-Wide Analysis of Basic Helix-Loop-Helix Transcription Factors to Elucidate Candidate Genes Related to Fruit Ripening and Stress in Banana (*Musa acuminata* L. AAA Group, cv. Cavendish)

**DOI:** 10.3389/fpls.2020.00650

**Published:** 2020-05-27

**Authors:** Zhuo Wang, Caihong Jia, Jing-Yi Wang, Hong-Xia Miao, Ju-Hua Liu, Cui Chen, Hui-Xiao Yang, Biyu Xu, Zhiqiang Jin

**Affiliations:** ^1^Key Laboratory of Tropical Crop Biotechnology, Ministry of Agriculture and Rural Affairs, Institute of Tropical Bioscience and Biotechnology, Chinese Academy of Tropical Agricultural Sciences, Hainan, China; ^2^Hainan Academy of Tropical Agricultural Resource, Chinese Academy of Tropical Agricultural Sciences, Hainan, China

**Keywords:** bHLH, banana, fruit ripening, stresses, co-expression networks, expression analysis

## Abstract

The basic helix−loop−helix (bHLH) proteins are a superfamily of transcription factors (TFs) that can bind to specific DNA target sites, playing a central role in a wide range of metabolic, physiological, and developmental processes in higher organisms. However, no systemic analysis of bHLH TFs has been reported in banana, a typical climacteric fruit in tropical and subtropical regions. In our study, 259 MabHLH TF genes were identified in the genome of *Musa acuminata* (A genome), and phylogenetic analysis indicated that these MabHLHs could be classified into 23 subfamilies with the bHLHs from rice and *Arabidopsis*. The amino acid sequences of the bHLH domain in all MabHLH protein sequences were quite conserved, especially Arg-12, Arg-13, Leu-23, and Leu-79. Distribution mapping results showed that 258 MabHLHs were localized on the 11 chromosomes in the *M*. *acuminata* genome. The results indicated that 40.7% of gene duplication events were located in collinear fragments, and segmental duplications might have played a key role in the expansion of *MabHLHs*. Moreover, the expression profiles of *MabHLHs* in different fruit development and ripening stages and under various abiotic and biotic stresses were investigated using available RNA-sequencing data to obtain fruit development, ripening-specific, and stress-responsive candidate genes. Finally, a co-expression network of *MabHLHs* was constructed by weighted gene co-expression network analysis to elucidate the *MabHLHs* that might participate in important metabolic biosynthesis pathways in banana during development and the response to stress. A total of 259 *MabHLHs* were identified, and their sequence features, conserved domains, phylogenetic relationships, chromosomal distributions, gene duplications, expression profiles, and co-expression networks were investigated. This study systematically identified the *MabHLHs* in the *M*. *acuminata* genome at the genome-wide level, providing important candidate genes for further functional analysis. These findings improve our understanding of the molecular basis of developmental and stress tolerance in an important banana cultivar.

## Introduction

Transcription factors (TFs) are important regulatory factors that interact with *cis-*elements to regulate the expression of genes in order to adapt to environmental stress in eukaryotes (Riechmann et al., 2000). Basic helix−loop−helix (bHLH) proteins contain a helix−loop−helix (HLH) structure (Kavas et al., 2016). The bHLH family is the second largest TF gene family (Feller et al., 2011) and plays an important regulatory role in the process of growth and development in plants (Pires and Dolan, 2010).

The typical bHLH domain usually contains 60 amino acids and can be divided into two functional regions: a basic area of 10–15 amino acids and a spiral area (HLH region) with 40 amino acids (Heim et al., 2003; Feller et al., 2011). The alkaline region is located at the N-end of the BHLH domain, adjacent to the HLH region, and the region is mainly associated with the binding of TFs and specific sequences. The HLH region is located in the C-end of the bHLH domain, containing two adjacent α-helices. The homologous or heterogeneous dimers of the α-helices from the same or different bHLH TFs can be combined with motifs in the promoter of the target gene, thereby regulating the expression of the target gene (Heim et al., 2003).

An increasing number of bHLH superfamily members have been functionally characterized in various plant species since the first plant bHLH gene, which plays a key role in anthocyanin biosynthesis, was isolated from *Zea mays* (Ludwig et al., 1989). As an increasing number of genome sequences are being released, a variety of bHLH superfamily genes have been identified and analyzed in a wide range of plant species, such as *Arabidopsis* (Carretero-Paulet et al., 2010), soybean (Hudson and Hudson, 2015), wheat (Wang L. et al., 2019), Chinese jujube (Li et al., 2019), rice (Li et al., 2006), peach (Zhang et al., 2018), Chinese cabbage (Song et al., 2014), and cotton (Lu et al., 2018). However, the classification of a large number of TFs in the bHLH family has proved challenging (Carretero-Paulet et al., 2010). In animals, bHLH proteins have been organized into six groups, A through F, based on the conservation of specific amino acids and the bHLH and other domains (Atchley and Fitch, 1997). In plants, the classification of bHLH proteins is usually based on sequence homology within the conserved bHLH domain, and the number of subgroups ranges from 15 to 26 (Buck and Atchley, 2003; Pires and Dolan, 2010). Phylogenetic analyses of some atypical bHLH proteins have even extended that number to 32 subgroups (Carretero-Paulet et al., 2010).

In plants, most of the functionally characterized genes originate from *Arabidopsis* (Carretero-Paulet et al., 2010). The multifunctional bHLH TFs participate in a wide range of plant growth and developmental processes (Nesi et al., 2000; Ramsay et al., 2003; Groszmann et al., 2008; Kondou et al., 2008; Fujisawa et al., 2013) and secondary metabolism (Ludwig et al., 1989; Bailey et al., 2003; Heim et al., 2003; Zhang et al., 2012). bHLH TF family members also play key roles in the response to environmental stresses, such as drought (Seo et al., 2011), salt (Zhou et al., 2009), low temperature (Wang Y. et al., 2012), and pathogen attack (Kim et al., 2012; Fan et al., 2014; Song et al., 2013). In banana, the study of bHLH is still in its infancy, and only a few bHLH genes have been functionally characterized (Peng et al., 2013; Zhao et al., 2013). The completion of the genome sequence of the *Musa acuminata* genome, a wild banana that belongs to subspecies *M*. *acuminata* ssp. *malaccensis*, has made a genome-wide analysis of bHLH genes possible (D’Hont et al., 2012).

Banana (*Musa*. spp.) is an important staple food for many people in subtropical and tropical regions. It is rich in protein and carbohydrates, and banana production contributes significantly to many people’s incomes (Aurore et al., 2009). Cavendish banana (*M*. *acuminata* AAA group cv. Cavendish) is a triploid (AAA) cultivar banana. However, the yield and quality of the banana fruits are often hindered by abiotic and biotic stresses, such as drought (van Asten et al., 2011), low temperature (Kang et al., 2007), salt (Xu et al., 2014), and several devastating diseases (Heslop-Harrison and Schwarzacher, 2007). One of the most important diseases, Panama disease or fusarium wilt of banana, is caused by the fungus *Fusarium oxysporum* f. sp. *cubense* (Foc) (Stover, 1962) and is widely regarded as one of the most destructive plant diseases in the world. Specifically, Foc tropical race 4 (Foc TR4) is a destructive disease of the commercial banana cultivar Cavendish (Ploetz, 2006; Visser et al., 2010). TR4 is responsible for most of the destruction to the commercial cultivar Cavendish, which is the main banana cultivar worldwide. Giant Cavendish Tissue Culture Variants (GCTCV) have acquired resistance to TR4 through somaclonal variation (Hwang and Ko, 2004). GCTCV-119 is the best Foc TR4-tolerant alternative cultivar for the Cavendish (Ploetz, 2015). Since the bHLH family has been proved to be associated with abiotic and biotic stress tolerance in many plant species, we systematically analyzed the bHLH gene family in banana to assess its potential relevance to fruit ripening and stress tolerance.

## Materials and Methods

### Identification MabHLH Genes in the *Musa acuminata* Genome and Phylogenetic Analyses

To analyze the genome-wide bHLH genes, we firstly downloaded all gene coding protein sequences of *M*. *acuminata* genome (A genome) DH Pahang v2 from the Banana Genome Hub^1^ (Martin et al., 2016). Subsequently, We used the iTAK program^16^ to identify TFs based on the consensus rules that are mainly summarized within PlnTFDB and PlantTFDB (Pérez-Rodríguez et al., 2009; Jin et al., 2013), and obtained all candidate MabHLH protein sequences. Finally, all candidate MabHLH protein sequences were further examined by the BLASTp and CDD^2^ databases in NCBI. The bHLH protein sequences from rice and *Arabidopsis* were acquired from the RGAP^3^ and TAIR^4^ databases, respectively. The full-length MabHLHs protein sequence from *M*. *acuminata*, *Arabidopsis*, and rice were aligned using ClustalW. Relationships were assessed using a bootstrap neighbor-joining evolutionary tree with 1,000 bootstrap replicates, created using MEGA 6.0 software (Tamura et al., 2013). The accession number of identified banana bHLHs is listed in Supplementary Table 1. The molecular weight and isoelectric points of the MabHLHs were predicted from the ExPASy database^5^. The sequence logo for bHLH domain was created by submitting the multiple alignment sequences to the WebLogo server^6^.

### Chromosome Distribution and Gene Duplications

To determine the physical locations of bHLH genes, the starting and ending positions of all bHLH genes on each chromosome were obtained from the banana A genome database. MapInspect software was used to draw the images of the locations of the banana bHLH genes^7^.

Tandem and segmental duplications were also identified according to the plant genome duplication database (Lee et al., 2012). Examples of tandem duplication were identified based on physical chromosomal location: homologous MabHLH genes on a single chromosome, with no other intervening genes, located within 30 kbp of each other, were characterized as tandem duplication (Shiu and Bleecker, 2003; Du et al., 2013). Syntenic blocks were detected using MCSCAN (parameters: -a -e 1e-5 -s 5) (Wang Z. et al., 2019), and all MabHLH genes located in the syntenic blocks were extracted. Circos (0.63) software was used to draw the images of the locations and synteny of the MabHLH genes^8^.

### Plant Materials and Treatments

Cavendish banana (*M*. *acuminata* L. AAA group cv. Cavendish) is a triploid cultivar characterized by a high yield, high quality, and long-term storage. However, this variety is highly susceptible to Foc TR4. The banana fruits were obtained from the Banana Plantation of the Institute of Tropical Bioscience and Biotechnology (Chengmai, Hainan, 20N, 110E). For temporal expression analysis, developing banana fruits of 0, 20, and 80 DAF, representing fruit developmental stages of budding, cutting flower, and harvest stages, respectively, were collected from both banana varieties. BX fruits stored for 0, 8, and 14 DPH, representing the three progressive ripening stages based on color of the fruit, including green, yellowish green, and yellow, respectively, have been selected for post-harvest analysis. One month old banana seedlings were grown in Hoagland’s solution (0.51 g/l KNO_3_, 0.82 g/l Ca(NO_3_)_2_, 0.49 g/l MgSO_4_⋅7H_2_O, 0.136 g/l KH_2_PO_4_, 0.6 mg/l FeSO_4_, 2.86 mg/l H_3_BO_3_, 1.81 mg/l MnCl_2_⋅4H_2_O, 0.08 mg/L CuSO_4_⋅5H_2_O, 0.22 mg/l ZnSO_4_⋅7H_2_O, 0.09 mg/l H_2_MoO_4_⋅4H_2_O) (pH 6.0) under greenhouse conditions (Arnon and Hoagland, 1939). The minimum and maximum temperatures in the greenhouse during the experiment were 25°C and 30°C, respectively, while the relative humidity oscillated between 55 and 80%. For salt and osmotic treatments, banana seedlings were irrigated with 300 mmol⋅L^–1^ NaCl and 200 mmol⋅L^–1^ mannitol for 7 days and havest the leaves without major vein to analysis. For cold treatment, banana plants were maintained at 4°C for 22 h and havest the leaves without maine vein to analysis. For Foc TR4 treatments, we used the susceptible cultivar Cavendish banana and resistant cultivar GCTCV-119 (*M*. *acuminata* L. AAA group, cv. GCTCV-119) to inoculate Foc TR4 (Hwang and Ko, 2004). The roots of five-leaf stage banana seedlings were dipped in a Foc TR4 spore suspension of 1.5 × 10^6^ condia/mL. The entire root system was then harvested at 0, 2, 4, and 6 days post-infection (DPI) (Wang Z. et al., 2012). All of the above samples were immediately frozen in liquid nitrogen and stored at −80°C until RNA extraction for transcriptome analysis and QRT-PCR verification.

### Transcriptomic Analysis

Total RNAs were isolated using a plant RNA extraction kit (TIANGEN, Beijing, China). Three μg of total RNA from each sample was converted to cDNA using a RevertAid First-Strand cDNA Synthesis Kit (Fermentas, Beijing, China). cDNA libraries were constructed using TruSeq RNA Library Preparation Kit v2, and were subsequently sequenced on an Illumina HiSeq 2,000 platform (San Diego, CA, United States) using the Illumina RNA-seq protocol. Two biological replicates were used for each sample. Gene expression levels were calculated as Reads Per Kilobases per Million reads (RPKM) (Mortazavi et al., 2008). Differentially expressed genes were identified with the read count of two replicates for each gene (fold change ≥ 2; FDR ≤ 0.001) (Audic and Claverie, 1997). A heat-map was constructed with MeV 4.9 and Java Treeview software according to the manufacturer’s protocol. All the data of RNA-seq has been uploaded to deposit in the CNSA^9^ of CNGBdb with accession number CNP0000292 (CNX0051086, CNX0051087, CNX0051090, CNX0051091, CNX0051097, CNX0051098, CNX0051099, CNX0051103, CNX0051104, CNX0051108, and CNX0051109) and analyzed as described in a previous study (Wang Z. et al., 2019).

### Weighted Gene Co-expression Network Analysis

Gene expression patterns for all identified genes were used to construct a co-expression network using WGCNA (version 1.47) (Langfelder and Horvath, 2008). Genes without expression detected in all tissues were removed prior to analyses. Soft thresholds were set based on the scale-free topology criterion employed by Zhang and Horvath (2005). An adjacency matrix was developed using the squared Euclidean distance values, and the topological overlap matrix was calculated for unsigned network detection using the Pearson method. Co-expression coefficients more than 0.55 between the target genes were then selected. Finally, we extracted the co-expression network of all *MabHLHs* and the network connections were visualized using cytoscape (Shannon et al., 2003).

### Quantitative RT-PCR (QRT-PCR) Analysis

Total RNA was isolated from banana roots before treatment. First-strand cDNA was synthesized from 2 μg poly-(A)^+^ RNA from each sample using AMV Reverse Transcriptase. SYBR Premix Ex Taq (TaKaRa, Dalian, China) was used in 25 μL reactions with 0.5 μL ROX reference dye. Primers (100 nM each) were mixed with the equivalent of 100 ng reverse-transcribed RNA template per reaction. In all experiments, negative controls containing no template RNA were subjected to the same procedure to exclude or detect any possible contamination. Before proceeding with the actual experiments, a series of template dilutions were performed to determine the optimal template concentration necessary to obtain the maximal amplification of the target.

Each qRT-PCR was performed on a Stratagene Mx3000P (Stratagene, CA, United States) machine using SYBR chemistry. The thermal cycling conditions were as follows: 94°C for 3 min, followed by 40 cycles of 94°C for 15 s, 55°C for 20 s, and 72°C for 40 s. Reactions were performed in triplicate, and data were analyzed using MxProTM QPCR software (Stratagene, CA, United States). The *MaActin* transcript (Genebank accession numbers: EF672732) was used as a control. All the primers are listed in Supplementary Table 12, and the experiments were carried out with three biological replicates. The differences in C_*t*_ values between the *MabHLHs* and *MaActin* transcripts were expressed as fold-changes related to *MaActin*.

### Statistical Analysis

Statistical analysis was performed using Student’s *t*-test. The experimental results obtained were expressed as the means ± standard deviation (SD). *P* values < 0.05 were considered statistically significant (^∗^), and *P* values < 0.01 were considered highly statistically significant (^∗∗^).

## Results

### Identification and Classification of MabHLHs in Banana

**FIGURE 1:**
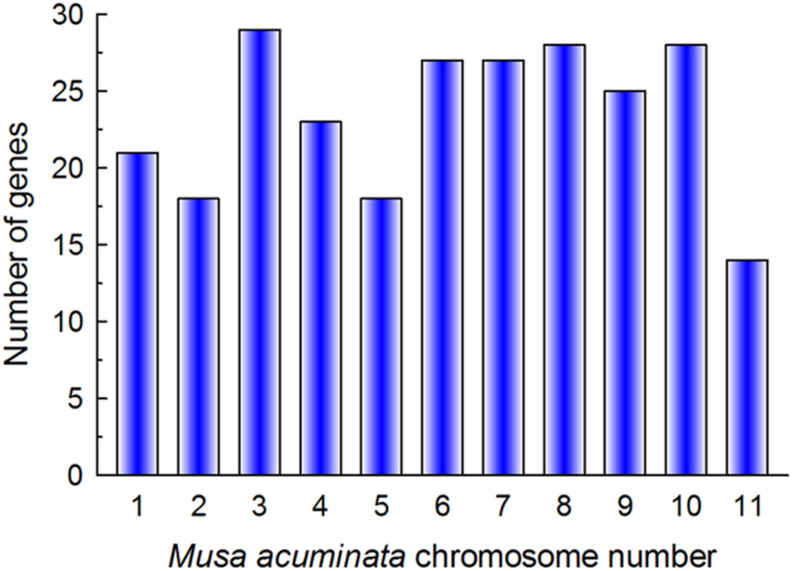
Gene numbers in 11 chromosomes of the *Musa acuminata* genome.

In total, 267 putative bHLH TFs were identified on the iTAK website (Zheng et al., 2016). The bHLH protein sequences encoded by non-representative transcripts were excluded. The remaining sequences were assessed for the existence of complete bHLH domains using the Conserved Domains Database (CDD). In total, 259 sequences were confirmed as putative members of the MabHLH family. The *MabHLHs* were randomly distributed on 11 chromosomes and were named *MabHLH001* to *MdbHLH258* based on their chromosomal locations. The highest number of MabHLH genes (29) was distributed on chromosome 3, while the least genes (14) were distributed on chromosome 11 (Figure 1). *Ma00_t02280*.*1* was not anchored on the chromosome and was named *MabHLH259*. The length of the MabHLH proteins varied from 85 (MabHLH061, MabHLH089, MabHLH099, MabHLH114, MabHLH132, MabHLH154, MabHLH195, and MabHLH222) to 712 (MabHLH077) amino acids. EXPASY analysis revealed that the MabHLH protein sequences had large variations in isoelectric point (*pI*) values (ranging from 4.56 to 10.34) and molecular weight (ranging from 10.36 to 79.67 kDa). The details regarding the location of the MabHLH protein sequences are summarized in Supplementary Table 1.

### Phylogenetic Analysis of the MabHLH Protein Family

**FIGURE 2:**
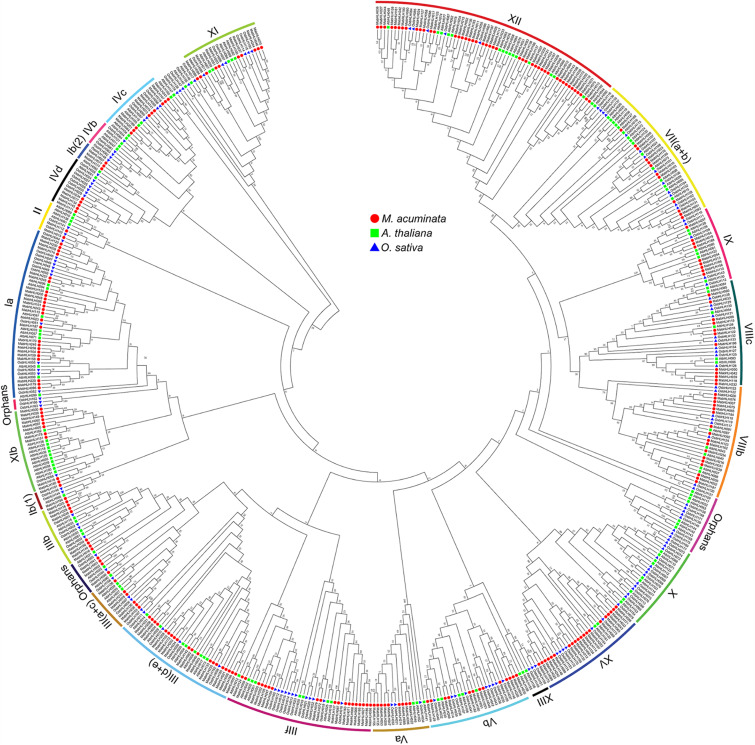
Phylogenetic analysis of the bHLHs from *Arabidopsis*, rice, and *M*. *acuminata* genome. The neighbor-joining (NJ) tree was drawn using MEGA 6.0 with 1,000 bootstrap replicates.

To analyze the evolutionary relationships among the MabHLHs, 259 MabHLH proteins were aligned with 147 AtbHLH proteins from *Arabidopsis* (Li et al., 2006) and 157 OsbHLH proteins from rice (Pires and Dolan, 2010), and an unrooted phylogenetic tree was constructed by MEGA6 (Supplementary Table 2). The 259 MabHLHs were grouped into 23 subfamilies. In banana, there was no MabHLH member distributed in the subfamilies X, IIIX, and IVb. Furthermore, four bHLH proteins (MabHLH002, MabHLH003, MabHLH041, and MabHLH098) were not distributed in any subgroups, and were termed ‘orphans’ subgroups (Pires and Dolan, 2010). There were 45 MabHLHs distributed in subgroup XII, which was the largest; 25 MabHLHs distributed in subgroup Ia; and 20 MabHLHs distributed in the subgroup IIId + e and XV subfamilies. In these subgroups, the number of MabHLH members was far greater than that of *Arabidopsis* and rice, suggesting the significant expansion of MabHLHs in banana (Figure 2).

### Conserved Amino Acid Residues in the MabHLHs Domain

**FIGURE 3:**
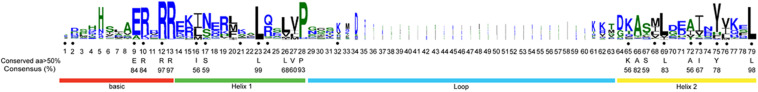
bHLH domain is highly conserved across all MabHLH proteins. The overall height of each stack represents the conservation of the sequence at that position. The black dot indicates the position of the 19 conserved amino acids previously identified by Atchley and Fitch (1997), and capital letters indicate over 50% conservation of amino acids among the 259 MabHLH domains.

Using CD-Search in NCBI and ClustalW align, all 259 protein sequences were found to contain the bHLH domain. The longest conserved domain was composed of 79 amino acids, including one alkaline region (basic) and one HLH region (helix 1) at the N-terminal, another HLH region (helix 2) at the C-terminal, and a loop located between helix 1 and helix 2, which was the least conserved (Figure 3). The frequencies of the 18 amino acid sites were higher than 50%. Four amino acid sites (Arg-12, Arg-13, Leu-23, and Leu-77) were conserved, and the frequencies of the conserved amino acids exceeded 95%. The frequencies of nine amino acid sites (Glu-9, Arg-10, Arg-12, Arg-13, Leu-23, Pro-28, Ala-66, Leu-83, and Leu-79) were higher than 80%. All of the above amino acid sites were located in the basic, helix 1, and helix 2 regions. We found that the two helix regions were most conserved, followed by the basic region and then the loop region. Between the two helix regions, the most conserved site was located in helix 1 (Leu-23, 99%) (Figure 3).

### Chromosomal Localization and Gene Duplication of MabHLHs

**FIGURE 4:**
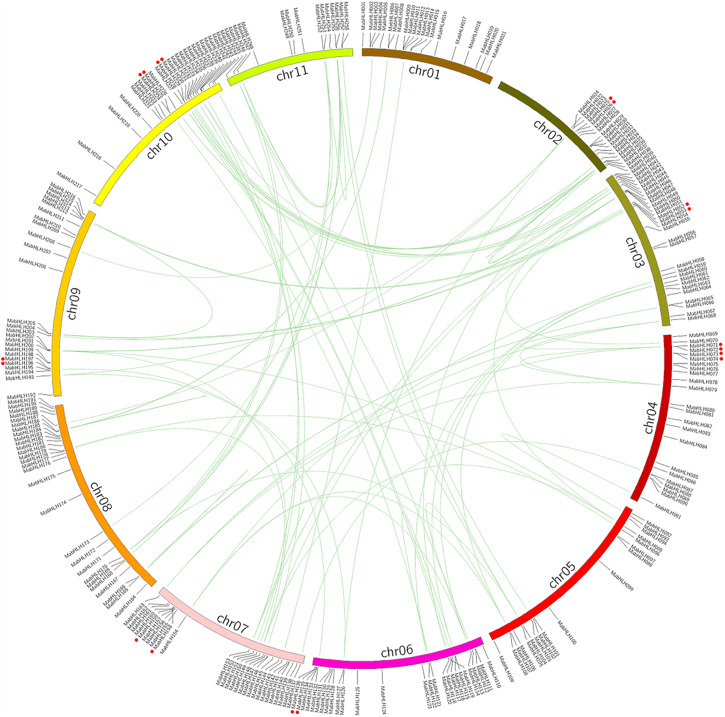
Distribution and synteny analysis of *MabHLHs* on the 11 chromosomes of *M*. *acuminata* genome. The locations of the MabHLH genes are indicated by vertical black lines. The segmental duplicate MabHLH genes are connected with green lines, and red dot indicates tandem duplicated MabHLH genes.

Genome chromosomal location analyses revealed that 258 *MabHLHs* were distributed on 11 chromosomes. As shown in Figure 1, the number of *MabHLHs* was irregular, although the *MabHLHs* were distributed on all of the 11 chromosomes. Although each of the 11 banana chromosomes contained *MabHLHs*, the distribution appeared to be uneven. There was no member located in the upper end of chromosome 2 (Figure 4). The distribution of *MabHLHs* on individual chromosomes also indicated certain physical regions with a relatively higher accumulation of gene clusters. For example, the *MabHLHs* on chromosomes 3, 6, 7, 8, 9, and 10 appeared to be congregated at the lower end and upper end of the arms (Figure 4).

Segmental duplication, tandem duplication, and retrotransposition are known to be key factors driving gene family expansion (Hurles, 2004; Freeling, 2009). Based on the segmental fragment information in the *M*. *acuminata* genome (D’Hont et al., 2012), 92 colinear *MabHLHs* representing approximately 35.6% (92 of 258) of the total *MabHLHs* were found to be located in the syntenic blocks and had been segmentally duplicated (Figure 4 and Supplementary Table 3). Interestingly, we found that all the colinear *MabHLHs* within the syntenic regions belonged to the same group in the phylogenetic tree (Figure 2). By locating the genes on the chromosome, nine pairs were identified as tandemly duplicated (Figure 4). We did not find any retrotransposons. Based on our analysis, we propose that the expansion of *MabHLHs* was mainly via segmental duplication during the *M*. *acuminata* genome evolutionary process.

### Expression Profile of *MabHLHs* During Fruit Development and the Postharvest Ripening Stage

**FIGURE 5:**
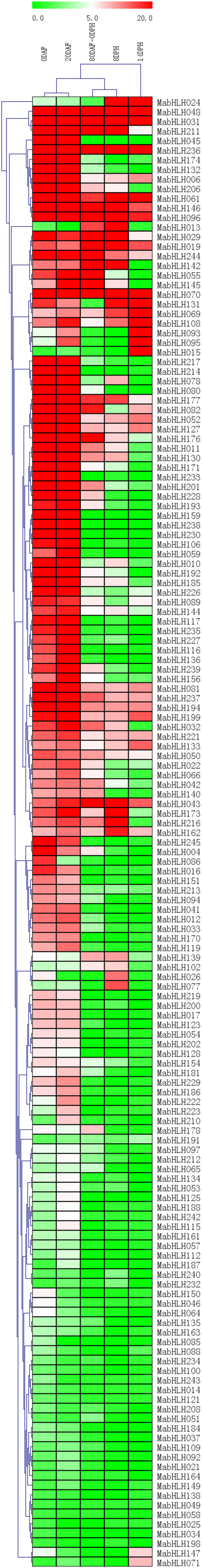
Expression patterns of *MabHLHs* in different stages of fruit development and ripening. The heatmap with dendrogram was created based on the RPKM value of the *MabHLHs*. Differences in gene expression changes are shown in color as the scale. Days after flowering (DAF) are fruit development stages, while days post-harvest (DPH) are fruit ripening stages.

To analyze the expression profiles of *MabHLHs*, RNA-Seq data were derived from the fruit development and ripening stages. We deleted 110 *MabHLHs* with Reads Per Kilobase of transcript per Million mapped reads (RPMK) values less than 1 in 0DAF, 20DAF, 80DAF_0DPH, 8DPH, and 14DPH. There were 97 and 95 *MabHLHs* highly expressed at 0 days after flowering (DAF) and 20 DAF (RPKM > 5), contributing about 37.5% and 36.7% of the total, respectively. We found that 51, 40, and 30 *MabHLHs* were highly expressed at 80 DAF–0 days post harvesting (DPH), 8 DPH, and 14 DPH (RPKM > 5), only contributing about 19.7, 15.4, and 11.6% of the total, respectively (Figure 5 and Supplementary Table 5). These results indicated that the *MabHLHs* are mainly involved in the development of banana fruit. Among them, *MabHLH019*, *MabHLH031*, *MabHLH048*, *MabHLH061*, *MabHLH070*, *MabHLH096*, *MabHLH146*, and *MabHLH236* were highly constitutively expressed at 0 DAF, 20 DAF, 80 DAF-0 DPH, 8 DPH, and 14 DPH (Figure 5 and Supplementary Table 5), suggesting that these genes play regulatory roles at the fruit developmental and postharvest ripening stages.

### The Expression Level of *MabHLHs* in the Banana Seedlings in Response to Osmotic, Salt, Cold, and Foc TR4 Treatments

**FIGURE 6:**
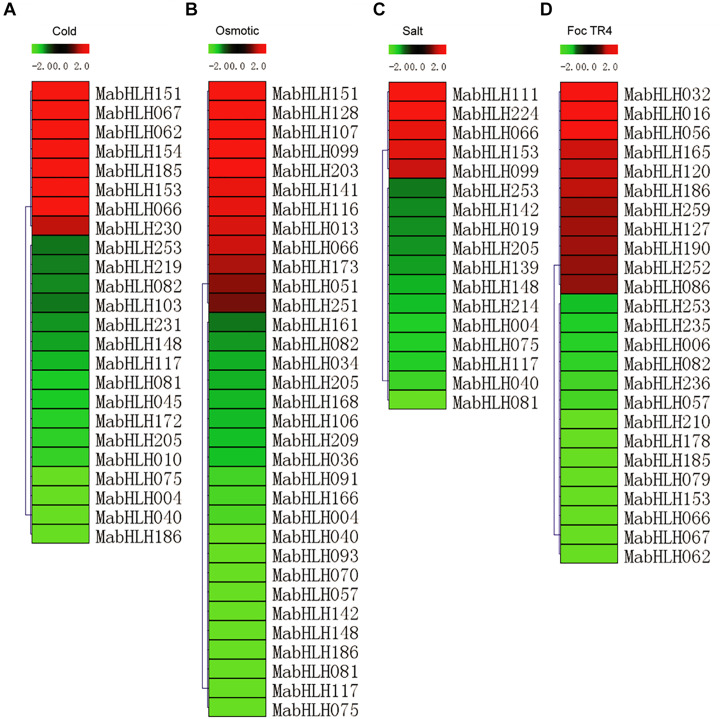
Expression patterns of *MabHLHs* in response to cold **(A)**, osmotic **(B)**, and salt **(C)** treatments and inoculation with Foc TR4 **(D)** in banana. The Log2-based fold change was used to create the heatmap. Differences in gene expression changes are shown in color as the scale.

To analyze the expression profiles of the *MabHLHs*, RNA-Seq data were derived from banana seedlings in response to osmotic, salt, cold, and Foc TR4 treatments. To present the differentially expressed genes visually and exactly, we filtered out the genes with RPKM values less than 5 in both the control (0 day post infection) and treatments. Low-temperature treatment (4°C) caused the 24 *MabHLHs* to be differentially expressed (log2 Ratio Cold/Control > 1), among which eight were up-regulated and 16 were down-regulated (Figure 6A and Supplementary Table 6). Treatment with drought (200 mmol⋅L^–1^ mannitol) caused the 33 *MabHLHs* to be differentially expressed (log2 Ratio Osmotic/Control > 1), among which 12 were up-regulated and 21 were down-regulated (Figure 6B and Supplementary Table 7). Treatment with salt (300 mmol⋅L^–1^ NaCl) caused 17 *MabHLHs* genes to be differentially expressed (log2 Ratio Salt/Control > 1), including five that were up-regulated and 12 that were down-regulated (Figure 6C and Supplementary Table 8). Infection with Foc TR4 resulted in the differential expression of 25 *MabHLHs* (log2 Ratio 2 DPI/0 DPI > 1), including 11 up-regulated and 14 down-regulated genes (Figure 6D and Supplementary Table 9). These results suggest that these differentially expressed *MabHLHs* may play important roles in the response of banana to Foc TR4 infection. In the above osmotic, salt, cold, and Foc TR4 infection treatments, we detected 67 differentially expressed *MabHLHs* in banana, accounting for only 25.86% of the total. Generally, we found that most of the banana bHLH TFs were not involved in the response to various abiotic and biotic stresses. The 67 differentially expressed *MabHLHs* detected above may play important roles in the response to multiple stresses in banana.

### Weighted Gene Co-expression Network of *MabHLHs*

**FIGURE 7:**
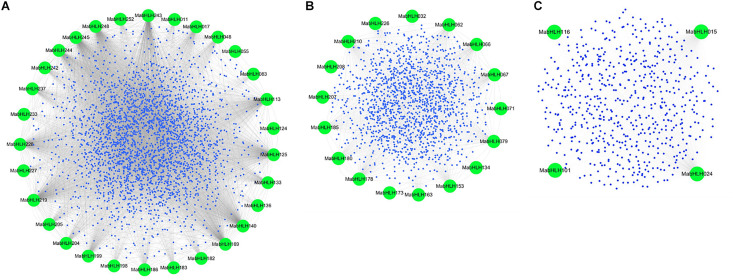
Co-expression network of banana generated using the *MabHLHs* as guides. The network comprises 4761 genes (nodes); the green nodes represent *MabHLHs*; and all other genes are depicted as small blue nodes. **(A)** Model 1, **(B)** Model 2, and **(C)** Model 3.

Co-expression networks provide insights into the patterns of transcriptome organization and suggest common biological functions for networked genes. Weighted Gene Co-Expression Network Analysis (WGCNA) is a method frequently used to explore the complex relationships between genes and phenotypes. The distinct advantage is that WGCNA transforms gene expression data into co-expression modules, providing insight into signaling networks that may be responsible for phenotypic traits of interest (Horvath et al., 2006; Shi et al., 2010; Udyavar et al., 2013). To explore the functions, 259 *MabHLHs* were selected as “guide genes” to seek co-expressed genes using an RNA-Seq dataset from 11 different transcriptomes, including fruit development and ripening stages, banana seedling response to osmotic, salt, and cold treatment, and banana roots inoculated with Foc TR4 (Supplementary Table 4). A total of 4761 genes as the target node, whose expression patterns were closely correlated with 48 *MabHLHs*, were identified with weighted values larger than 0.5 (Supplementary Table 9). Following visualization using Circos, the co-expression network of *MabHLHs* was divided into three models. There were 30, 17, and three *MabHLHs* contained in models 1−3, respectively (Figure 7 and Supplementary Table 10).

**FIGURE 8:**
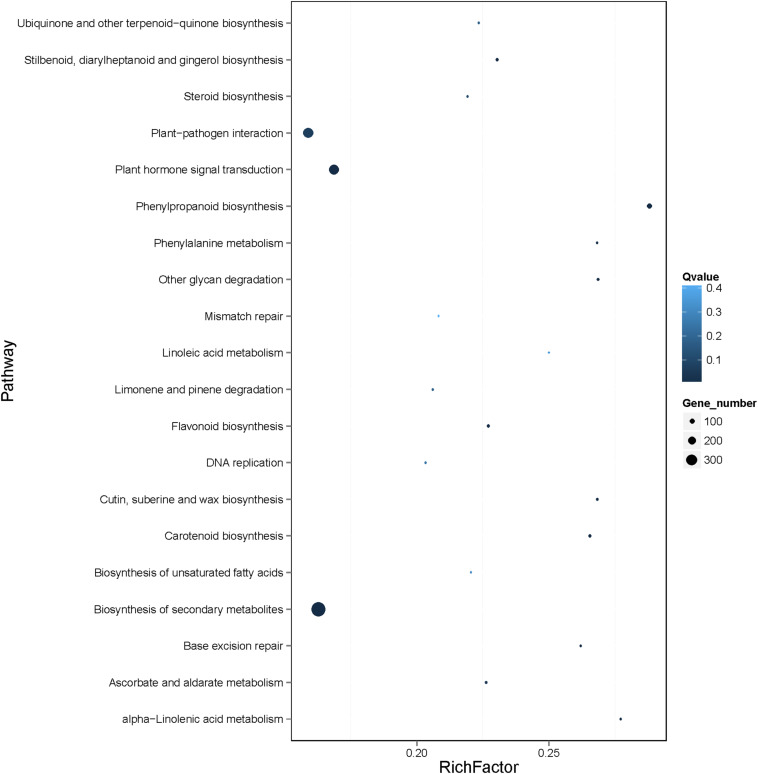
KEGG pathway enrichment analysis of the genes in the co-expression network of the MabHLH genes. A total of 4809 (4761 target node genes and 48 *MabHLHs*) genes were used in the enrichment analysis. The gene set enrichment was analyzed using hypergeometric testing. The *Q*-value was calculated using the FDR (false discovery rate) adjustment method for correcting multiple hypothesis testing. The top 20 pathways are shown. *Q*-values represent the significance of the enrichment. Circles indicate the target genes, and the size is proportional to the number of genes.

According to Kyoto Encyclopedia of Genes and Genomes (KEGG) pathway enrichment analysis of the 4761 genes, 18 KEGG pathways were enriched, mainly including biosynthesis of secondary metabolites (ko01110), plant hormone signal transduction (ko04075), plant−pathogen interaction (ko04626), and phenylpropanoid biosynthesis (ko00940) (Figure 8 and Supplementary Table 11). Notably, the differentially expressed genes in banana in response to Foc TR4 infection, including *MabHLH032*, *MabHLH062*, *MabHLH066*, *MabHLH067*, *MabH LH079*, *MabHLH153*, *MabHLH178*, *MabHLH185*, *MabHLH186*, *MabHLH210*, and *MabHLH252*, were included in the weighted co-expression network of the *MabHLHs*. These results demonstrate that these *MabHLHs* might be important in regulating the response of banana to Foc TR4 infection.

### Expression Patterns of *MabHLHs* During the Interaction of Banana Plantlets With Foc TR4

We discovered that there were 11 *MabHLHs* involved in the above weighted co-expression network that were differentially expressed during the interaction of banana with Foc TR4, and all of them decreased significantly at 2 DPI in the RNA-Seq data. *MabHLH062*, *MabHLH066*, *MabHLH067*, *MabHLH079*, *MabHLH153*, and *MabHLH185* were highly expressed (RPKM value > 20) at 0 DPI (Supplementary Table 9).

**FIGURE 9:**
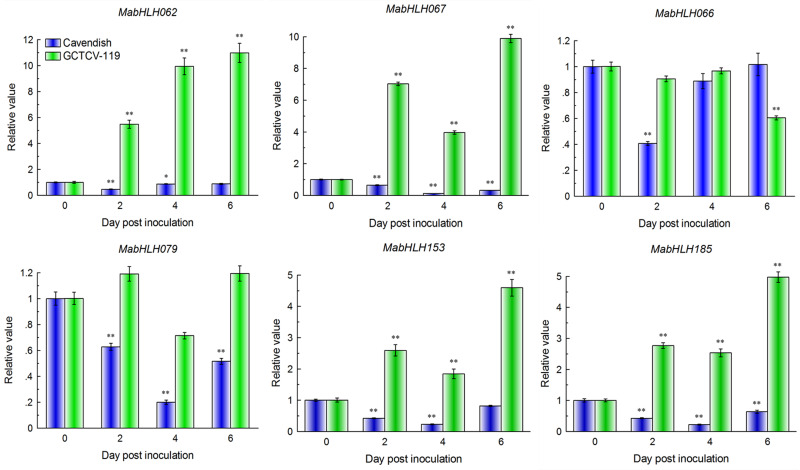
Expression patterns of six *MabHLH* genes in Cavendish and GCTCV-119 inoculated with Foc TR4 by qRT-PCR. The results are presented as differential relative transcript abundance. The data represent the mean ± standard deviation (SD) of three replicates. The *y*-axis shows the transcript fold-change relative to that in the control (0 DPI). * and **significantly different from the control (0 DPI) at *P* < 0.05 and 0.01, respectively.

These six differentially expressed *MabHLHs* were selected for quantitative real-time (qRT)-PCR analysis of their expression patterns in the response of the resistant (GCTCV-119) and susceptible (Cavendish) banana cultivars to Foc TR4 infection. RNA was extracted from the roots of two cultivars at 2 DPI, 4 DPI, and 6 DPI and subjected to qRT-PCR analysis. A mock treatment (0 DPI) was carried out using Hoagland’s solution as a control. In the Cavendish banana, all four *MabHLHs* decreased significantly *(P* < 0.05, the same below) at 2 DPI, 4 DPI, and 6 DPI. We also found that six *MabHLHs* showed the same trends and consistent results between the RNA-Seq data and qRT-PCR data at 0 DPI and 2 DPI (Figure 9). These results indicated that the RNA-Seq data were suitable for detecting the expression patterns of *MabHLHs*. Moreover, in the GCTCV-119 banana, *MabHLH062*, *MabHLH067*, *MabHLH153*, and *MabHLH185* increased significantly at 2 DPI and reached the highest relative expression levels of 10.98-fold, 9.88-fold, 4.59-fold, and 4.97-fold at 6 DPI, respectively. *MabHLH066* was not differentially expressed at 2 DPI and 4 DPI, and only decreased markedly at 6 DPI. *MabHLH079* was also not differentially expressed at all the time points (Figure 9). In summary, these results indicated that *MabHLH062*, *MabHLH067*, *MabHLH153*, and *MabHLH185* were involved in banana resistance to Foc TR4 infection in GCTCV-119 cultivar.

## Discussion

Whole-genome identification of bHLH genes may provide global insight into the basis for the functional diversification of these genes during evolution. The bHLH TFs comprise a large family in higher plants, and numerous studies have shown that bHLH TFs are involved in diverse biological processes in plant growth, development, and stress responses (Heim et al., 2003). In plants, bHLH is the second largest family of TFs and includes 319 members in soybean (Hudson and Hudson, 2015), 230 in Chinese cabbage (Song et al., 2014), 167 in rice (Li et al., 2006), and 162 in *Arabidopsis* (Carretero-Paulet et al., 2010). In our study, we identified 259 *MabHLHs* from the *M*. *acuminata* genome, which has been the second-highest number of bHLHs detected, to date. The characteristics of the number of encoding amino acids, isoelectric point, and molecular weight of the MabHLHs were similar to previous results in *Arabidopsis* and other plants (Carretero-Paulet et al., 2010).

Previous studies have shown that over 50% of plant bHLH proteins contain five alkaline amino acid residues in the conserved domain, as well as a highly conserved HER motif (His5-Glu9-Arg13), of which the predicted function is to bind with the target DNA motif (Atchley and Fitch, 1997; Massari and Murre, 2000; Toledo-Ortiz et al., 2003). We also found that all MabHLH proteins have conserved HER motifs. Compared with the conserved domain sequences of the HLH region from *Arabidopsis* (Feller et al., 2011), rice (Li et al., 2006), tomato (Sun et al., 2015), and Chinese cabbage (Song et al., 2014), we found a similar frequency of amino acid residues, and the amino acid residue sites in Leu-23, Leu-39, Tyr-45, and Leu-49 were highly conserved in the HLH region of the MabHLH proteins (Figure 3). We also found that the conservation of Helix1 was higher than that of Helix 2, which corroborates previous results in *Arabidopsis* (Feller et al., 2011), rice (Li et al., 2006), and tomato (Sun et al., 2015). Therefore, these findings indicated that the bHLH domain in the MabHLH proteins was highly conserved, and all members of MabHLHs are typical bHLH genes from the *M*. *acuminata* genome.

The *M*. *acuminata* genome has undergone three episodes of genome-wide duplication (WGD) events (α-, β-, and γ-WGD), and the duplicated segments included in the *Musa* ancestral blocks cover 222 Mb and contain 26,829 genes (D’Hont et al., 2012). We found that 92 *MabHLHs* were segmentally duplicated genes, and nine pairs of *MabHLHs* had been tandemly duplicated (Figure 4), which suggested that segmental duplication was the main pathway of *MabHLH* expansion in the *M*. *acuminata* genome.

Plant bHLHs play important roles in growth and development (Ding et al., 2009; Ikeda et al., 2013), stress tolerance (Kiribuchi et al., 2005; Miura et al., 2007; Long et al., 2010; Song et al., 2013; Fan et al., 2014), and signal transduction (Kiribuchi et al., 2005; Seo et al., 2011). In banana fruits, five *MabHLHs* were found to participate in methyl jasmonate (MeJA)-induced chilling tolerance (Peng et al., 2013), and *MaMYC2* belonging to the bHLH gene family can bind with *MaICE1* to regulate induced chilling tolerance in fruit (Zhao et al., 2013). In banana, *MabHLH6* was increased both in transcript and protein levels, and positively regulated the expression of starch degradation-related genes during fruit ripening process (Xiao et al., 2018). *MabHLH6* was negatively regulated by *MaMYB3*, which delayed the ripening of banana fruit (Fan et al., 2018). The transcript level of *MabHLH7* was obviously increased during banana fruit ripening, which was ethylene-inducible and nuclear-localized (Song et al., 2020). In our results, the expression of *MabHLH 024* (same with *MabHLH7*) and *MabHLH139* (same with *MabHLH6*) was also increased during 80DAF_0DPH, 8DPH and 14DPH (Figure 5), which is consistent with *MabHLH6* (Xiao et al., 2018; Song et al., 2020). Furthermore, about 40% of *MabHLHs* were highly expressed during banana fruit development (0 DAF and 20 DAF). The expression profile results indicated that the *MabHLHs* were significantly involved in fruit development. Banana fruit is a typical climacteric fruit that releases a large amount of ethylene during the postharvest ripening process (Liu et al., 1999). We found that most of the *MabHLHs* were lowly (RPKM < 5) or not expressed in the ripening stage, indicating that most of the *MabHLHs* may not be involved in the postharvest ripening of banana fruit. At the same time, we found that *MabHLH229*, *MabHLH035*, *MabHLH152*, *MabHLH263*, *MabHLH155*, and *MabHLH157* were differentially expressed and gradually decreased at 0 DPH, 8 DPH, and 14 DPH, suggesting that these genes might play negative regulatory roles in banana fruit during the postharvest ripening stage.

Many studies have shown that bHLH is involved in the tolerance to abiotic stress. In rice, *OsbHLH006* and *OsbHLH148* participate in the traumatic response and drought stress through regulating the expression of genes related to the rice jasmonic acid signaling pathway (Kiribuchi et al., 2005; Seo et al., 2011). In *Arabidopsis*, *AtICE1* TFs participate in plant stomatal development and low temperature stress (Miura et al., 2007; Kanaoka et al., 2008). In our results, we found that more than 20% of genes were differentially expressed in the cold, drought, and salt stress treatments. These results indicate that *MabHLHs* play important roles in the banana response to various abiotic stresses, such as drought, high salinity, and low temperature.

The bHLH TF family also plays an important role in plant disease resistance. In rice, *OsRAI1* encodes a putative basic helix–loop–helix TF. The expression of the *OsRAI1* gene can be induced by pathogens and can promote the expression of *OsPAL1* and *OsWRKY19* to participate in the defense response to rice blast (Kim et al., 2012). In *Arabidopsis*, *AtHBI1* (homolog of BEE2 interacting with IBH1OMOLOG OF BEE2 INTERACTING WITH IBH1) can form a basic complex with the PRE gene and IBH1 gene (PRE-IBH1-HBI1), playing a regulatory role in plant resistance to pathogen infection and growth (Fan et al., 2014). Members of the third bHLH TF subgroup, which includes *AtbHLH3*, *AtbHLH13*, *AtbHLH14*, and *AtbHLH17*, act as transcription repressors to interact with jasmonate ZIM-domain proteins to repress jasmonate responses (Song et al., 2013). In our results, we found that six *MabHLH* genes were highly expressed (RPKM > 20) in the banana roots during Foc TR4 infection (Figure 9 and Supplementary Table 9). Traditional biological research starts with a single gene or protein, which can only explain part of the system and makes it difficult to comprehensively explore the whole system. Gene networks are valuable for gene function discovery and candidate gene prioritization. Compared with other methods, WGCNA can better preserve the characteristics of biological networks, and the modules can better reflect the relationship among functions and different biological processes (Kadarmideen and Watson-Haigh, 2012). In the co-expression network, the plant−pathogen interaction (ko04626) pathway was enriched, indicating that *MabHLHs* might be involved in the banana response to Foc TR4 infection. We also found that 11 differentially expressed *MabHLHs* were contained in the co-expression network. In our study, the relative expression values of *MabHLH062*, *MabHLH067*, *MabHLH153*, and *MabHLH185* were larger in the resistant cultivar than in the susceptible cultivar (Figure 9). These results indicated that these *MabHLHs* were involved in banana resistance to Foc TR4 infection and the mutation site in GCTCV-119 cultivar selected by somatic variation might directly or indirectly affect the expression of these *MabHLHs* in the banana response to Foc TR4 infection.

## Conclusion

We systematically identified 259 *MabHLHs* in the *M*. *acuminata* genome. A total of 258 *MabHLHs* were located on 11 different chromosomes and could be classified into 23 main groups based on phylogenetic analysis with bHLHs from *Arabidopsis* and rice. We analyzed the expression profiles of the *MabHLHs* at different stages of fruit development and ripening and found numerous *MabHLHs* that participate in the banana response to abiotic and biotic stresses. Finally, the co-expression network of *MabHLHs* was constructed using WGCNA to elucidate the *MabHLHs* that might participate in important metabolic biosynthesis pathways in banana. The qRT-PCR results suggested that *MabHLH062*, *MabHLH067*, *MabHLH153*, and *MabHLH185* were involved in banana resistance to Foc TR4 infection. This comprehensive study improves our understanding of the *MabHLHs* associated with fruit development, ripening processes, and stress responses and will establish a foundation for future studies on genetic improvement in banana.

## Data Availability Statement

The datasets generated for this study can be found in the data has been uploaded to deposit in the CNSA (https://db.cngb.org/cnsa/) of CNGBdb with accession number CNP0000292.

## Author Contributions

ZW, BX, and ZJ conceived the study. ZW, CJ, J-YW, J-HL, CC, and H-XY analyzed the bHLH gene’s data from *M*. *acuminata* genome and performed the bioinformatics analysis. ZW, CJ, CC, and H-XY performed the Foc TR4 inoculation, RNA isolation, and qRT-PCR. ZW wrote the manuscript. All authors read and approved the final manuscript.

## Conflict of Interest

The authors declare that the research was conducted in the absence of any commercial or financial relationships that could be construed as a potential conflict of interest.

## Footnotes

1 https://banana-genome-hub.southgreen.fr

2 http://www.ncbi.nlm.nih.gov/cdd/

3 http://rice.plantbiology.msu.edu/

4 http://www.arabidopsis.org/

5 http://expasy.org/

6 http://weblogo.berkeley.edu/logo.cgi

7 http://mapinspect.software.informer.com/

8 http://circos.ca/

9 https://db.cngb.org/cnsa/

## References

[B1] Arnon D. I. Hoagland D. R. (1939). A comparison of water culture and soil as media for crop production. *Science* 89 512–514. 10.1126/science.89.2318.512 17776587

[B2] Atchley W. R. Fitch W. M. (1997). A natural classification of the basichelix-loop-helix class of transcription factors. *Proc. Natl. Acad. Sci. U.S.A.* 94 5172–5176. 10.1073/pnas.94.10.5172 9144210 PMC24651

[B3] Audic S. Claverie J. M. (1997). The significance of digital gene expression profiles. *Genome Res.* 7 986–995. 10.1101/gr.7.10.986 9331369

[B4] Aurore G. Parfait B. Fahrasmane L. (2009). Bananas, raw materials for making processed food products. *Trends Food Sci. Technol.* 20 78–91.

[B5] Bailey P. C. Martin C. Toledo-Ortiz G. Quail P. H. Huq E. Heim M. A. (2003). Update on the basic helix-loop-helix transcription factor gene family in *Arabidopsis thaliana*. *Plant Cell* 15 2497–2502. 10.1105/tpc.151140 14600211 PMC540267

[B6] Buck M. J. Atchley W. R. (2003). Phylogenetic analysis of plant basic helixloop-helix proteins. *J. Mol. Evol.* 56 742–750. 10.1007/s00239-002-2449-3 12911037

[B7] Carretero-Paulet L. Galstyan A. Roig-Villanova I. Martínez-García J. F. Bilbao-Castro J. R. Robertson D. L. (2010). Genome-wide classification and evolutionary analysis of the bHLH family of transcription factors in *Arabidopsis*, poplar, rice, moss, and algae. *Plant Physiol.* 153 1398–1412. 10.1104/pp.110.153593 20472752 PMC2899937

[B8] D’Hont A. Denoeud F. Aury J. M. Baurens F. C. Carreel F. Garsmeur O. (2012). The banana (*Musa acuminata*) genome and the evolution of monocotyledonous plants. *Nature* 448 213–217. 10.1038/nature11241 22801500

[B9] Ding W. Yu Z. Tong Y. Huang W. Chen H. Wu P. (2009). A transcription factor with a bHLH domain regulates root hair development in rice. *Cell Res.* 19:1309 10.1038/cr.2009.109 19752888

[B10] Du D. Zhang Q. Cheng T. Pan H. Yang W. Sun L. (2013). Genome-wide identification and analysis of late embryogenesis abundant (LEA) genes in *Prunus mume*. *Mol. Biol. Rep.* 40 1937–1946. 10.1007/s11033-012-2250-3 23086279

[B11] Fan M. Bai M. Y. Kim J. G. Wang T. Oh E. Chen L. (2014). The bHLH transcription factor HBI1 mediates the trade-off between growth and pathogen-associated molecular pattern–triggered immunity in *Arabidopsis*. *Plant Cell* 26 828–841. 10.1105/tpc.113.121111 24550223 PMC3967043

[B12] Fan Z. Q. Ba L. J. Shan W. Xiao Y. Y. Lu W. J. Kuang J. F. (2018). A banana R2R3-MYB transcription factor MaMYB3 is involved in fruit ripening through modulation of starch degradation by repressing starch degradation-related genes and MabHLH6. *Plant J.* 96 1191–1205. 10.1111/tpj.14099 30242914

[B13] Feller A. Machemer K. Braun E. L. Grotewold E. (2011). Evolutionary and comparative analysis of MYB and bHLH plant transcription factors. *Plant J.* 66 94–116. 10.1111/j.1365-313X.2010.04459.x 21443626

[B14] Freeling M. (2009). Bias in plant gene content following different sorts of duplication: tandem, whole-genome, segmental, or by transposition. *Annu. Rev. Plant Biol.* 60 433–453. 10.1146/annurev.arplant.043008.092122 19575588

[B15] Fujisawa M. Nakano T. Shima Y. Ito Y. (2013). A large-scale identification of direct targets of the tomato MADS box transcription factor RIPENING INHIBITOR reveals the regulation of fruit ripening. *Plant Cell* 25 371–386. 10.1105/tpc.112.108118 23386264 PMC3608766

[B16] Groszmann M. Paicu T. Smyth D. R. (2008). Functional domains of SPATULA,a bHLH transcription factor involved in carpel and fruit development in *Arabidopsis*. *Plant J.* 55 40–52. 10.1111/j.1365-313X.2008.03469.x 18315540

[B17] Heim M. A. Jakoby M. Werber M. Martin C. Weisshaar B. Bailey P. C. (2003). The basic helix-loop-helix transcription factor family in plants: a genome-wide study of protein structure and functional diversity. *Mol. Biol. Evol.* 20 735–747. 10.1093/molbev/msg088 12679534

[B18] Heslop-Harrison J. S. Schwarzacher T. (2007). Domestication, genomics and the future for banana. *Ann. Bot.* 100 1073–1084. 10.1093/aob/mcm191 17766312 PMC2759213

[B19] Horvath S. Zhang B. Carlson M. Lu K. V. Zhu S. Felciano R. M. (2006). Analysis of oncogenic signaling networks in glioblastoma identifies ASPM as a molecular target. *Proc. Natl. Acad. Sci. U.S.A.* 103 17402–17407. 10.1073/pnas.0608396103 17090670 PMC1635024

[B20] Hudson K. A. Hudson M. E. (2015). A classification of basic helix-loop-helix transcription factors of soybean. *Int. J. Genom.* 2015:603182 10.1155/2015/603182 PMC4339708 25763382

[B21] Hurles M. (2004). Gene duplication: the genomic trade in spare parts. *PLoS Biol.* 2:e206 10.1371/journal.pbio.0020206 PMC449868 15252449

[B22] Hwang S. C. Ko W. H. (2004). Cavendish banana cultivars resistant to Fusarium wilt acquired through somaclonal variation in Taiwan. *Plant Dis.* 88 580–588. 10.1094/PDIS.2004.88.6.580 30812575

[B23] Ikeda M. Mitsuda N. Ohme-Takagi M. (2013). ATBS1 INTERACTING FACTORs negatively regulate *Arabidopsis* cell elongation in the triantagonistic bHLH system. *Plant Signal. Behav.* 8:e23448. 10.4161/psb.23448 23333962 PMC3676513

[B24] Jin J. Zhang H. Kong L. Gao G. Luo J. (2013). PlantTFDB 3.0: a portal for the functional and evolutionary study of plant transcription factors. *Nucleic Acids Res.* 42 D1182–D1187. 10.1093/nar/gkt1016 24174544 PMC3965000

[B25] Kadarmideen H. N. Watson-Haigh N. S. (2012). Building gene co-expression networks using transcriptomics data for systems biology investigations: comparison of methods using microarray data. *Bioinformation* 8:855. 10.6026/97320630008855 23144540 PMC3489090

[B26] Kanaoka M. M. Pillitteri L. J. Fujii H. Yoshida Y. Bogenschutz N. L. Takabayashi J. (2008). SCREAM/ICE1 and SCREAM2 specify three cell-state transitional steps leading to *Arabidopsis* stomatal differentiation. *Plant Cell* 20 1775–1785. 10.1105/tpc.108.060848 18641265 PMC2518248

[B27] Kang G. Z. Wang Z. X. Xia K. F. Sun G. C. (2007). Protection of ultrastructure in chilling-stressed banana leaves by salicylic acid. *J. Zhejiang Univ. Sci. B* 8 277–282. 10.1631/jzus.2007.B0277 17444604 PMC1838828

[B28] Kavas M. Baloğlu M. C. Atabay E. S. Ziplar U. T. Daşgan H. Y. Ünver T. (2016). Genome-wide characterization and expression analysis of common bean bHLH transcription factors in response to excess salt concentration. *Mol. Genet. Genom.* 291 129–143. 10.1007/s00438-015-1095-6 26193947

[B29] Kim S. H. Oikawa T. Kyozuka J. Wong H. L. Umemura K. Kishi-Kaboshi M. (2012). The bHLH Rac Immunity1 (RAI1) is activated by OsRac1 via OsMAPK3 and OsMAPK6 in rice immunity. *Plant Cell Physiol.* 53 740–754. 10.1093/pcp/pcs033 22437844

[B30] Kiribuchi K. Jikumaru Y. Kaku H. Minami E. Hasegawa M. Kodama O. (2005). Involvement of the basic helix-loop-helix transcription factor RERJ1 in wounding and drought stress responses in rice plants. *Biosci. Biotechnol. Biochem.* 69 1042–1044. 10.1271/bbb.69.1042 15914931

[B31] Kondou Y. Nakazawa M. Kawashima M. Ichikawa T. Yoshizumi T. Suzuki K. (2008). RETARDED GROWTH OF EMBRYO1,a new basic helix-loop-helix protein, expresses in endosperm to control embryo growth. *Plant Physiol.* 147 1924–1935. 10.1104/pp.108.118364 18567831 PMC2492639

[B32] Langfelder P. Horvath S. (2008). WGCNA: an R package for weighted correlation network analysis. *BMC Bioinform.* 9:559. 10.1186/1471-2105-9-559 19114008 PMC2631488

[B33] Lee T. H. Tang H. Wang X. Paterson A. H. (2012). PGDD: a database of gene and genome duplication in plants. *Nucleic Acids Res.* 41 D1152–D1158. 10.1093/nar/gks1104 23180799 PMC3531184

[B34] Li H. Gao W. Xue C. Zhang Y. Liu Z. Zhang Y. (2019). Genome-wide analysis of the bHLH gene family in Chinese jujube (*Ziziphus jujuba* Mill.) and wild jujube. *BMC Genom.* 20:568. 10.1186/s12864-019-5936-2 31291886 PMC6617894

[B35] Li X. Duan X. Jiang H. Sun Y. Tang Y. Yuan Z. (2006). Genome-wide analysis of basic/helix-loop-helix transcription factor family in rice and *Arabidopsis*. *Plant Physiol.* 141 1167–1184. 10.1104/pp.106.080580 16896230 PMC1533929

[B36] Liu X. Shiomi S. Nakatsuka A. Kubo Y. Nakamura R. Inaba A. (1999). Characterization of ethylene biosynthesis associated with ripening in banana fruit. *Plant Physiol.* 121 1257–1265. 10.1104/pp.121.4.1257 10594112 PMC59492

[B37] Long T. A. Tsukagoshi H. Busch W. Lahner B. Salt D. E. Benfey P. N. (2010). The bHLH transcription factor POPEYE regulates response to iron deficiency in *Arabidopsis* roots. *Plant Cell* 22 2219–2236. 10.1105/tpc.110.074096 20675571 PMC2929094

[B38] Lu R. Zhang J. Liu D. Wei Y. Wang Y. Li X. (2018). Characterization of bHLH/HLH genes that are involved in brassinosteroid (BR) signaling in fiber development of cotton (*Gossypium hirsutum*). *BMC Plant Biol.* 18:304. 10.1186/s12870-018-1523-y 30482177 PMC6258498

[B39] Ludwig S. R. Habera L. F. Dellaporta S. L. Wessler S. R. (1989). Lc, a member of the maize R gene family responsible for tissue-specific anthocyanin production, encodes a protein similar to transcriptional activators and contains the myc-homology region. *Proc. Natl. Acad. Sci. U.S.A.* 86 7092–7096. 10.1073/pnas.86.18.7092 2674946 PMC298000

[B40] Martin G. Baurens F. C. Droc G. Rouard M. Cenci A. Kilian A. (2016). Improvement of the banana “*Musa acuminata*” reference sequence using NGS data and semi-automated bioinformatics methods. *BMC Genomics* 17:243 10.1186/s12864-016-2579-4 PMC4793746 26984673

[B41] Massari M. E. Murre C. (2000). Helix-loop-helix proteins: regulators of transcription in eucaryotic organisms. *Mol. Cell. Biol.* 20 429–440. 10.1128/mcb.20.2.429-440.2000 10611221 PMC85097

[B42] Miura K. Jin J. B. Lee J. Yoo C. Y. Stirm V. Miura T. (2007). SIZ1-mediated sumoylation of ICE1 controls CBF3/DREB1A expression and freezing tolerance in *Arabidopsis*. *Plant Cell* 19 1403–1414. 10.1105/tpc.106.048397 17416732 PMC1913760

[B43] Mortazavi A. Williams B. A. McCue K. Schaeffer L. Wold B. (2008). Mapping and quantifying mammalian transcriptomes by RNA-Seq. *Nat. Methods* 5 621–628. 10.1038/nmeth.1226 18516045 PMC13303166

[B44] Nesi N. Debeaujon I. Jond C. Pelletier G. Caboche M. Lepiniec L. (2000). The TT8 gene encodes a basic helix-loop-helix domain protein required for expression of DFR and BAN genes in *Arabidopsis* siliques. *Plant Cell* 12 1863–1878. 10.1105/tpc.12.10.1863 11041882 PMC149125

[B45] Peng H. H. Shan W. Kuang J. F. Lu W. J. Chen J. Y. (2013). Molecular characterization of cold-responsive basic helix-loop-helix transcription factors MabHLHs that interact with MaICE1 in banana fruit. *Planta* 238 937–953. 10.1007/s00425-013-1944-7 23955147

[B46] Pérez-Rodríguez P. Riano-Pachon D. M. Corrêa L. G. G. Rensing S. A. Kersten B. Mueller-Roeber B. (2009). PlnTFDB: updated content and new features of the plant transcription factor database. *Nucleic Acids Res.* 38 D822–D827. 10.1093/nar/gkp805 19858103 PMC2808933

[B47] Pires N. Dolan L. (2010). Origin and diversification of basic-helix-loop-helix proteins in plants. *Mol. Biol. Evol.* 27 862–874. 10.1093/molbev/msp288 19942615 PMC2839125

[B48] Ploetz R. C. (2006). Fusarium wilt of banana is caused by several pathogens referred to as *Fusarium oxysporum* f. sp. cubense. *Phytopathology* 96 653–656. 10.1094/PHYTO-96-0653 18943184

[B49] Ploetz R. C. (2015). Fusarium wilt of banana. *Phytopathology* 105:1512. 10.1094/PHYTO-04-15-0101-RVW 26057187

[B50] Ramsay N. A. Walker A. R. Mooney M. Gray J. C. (2003). Two basic-helix-loop-helix genes (MYC-146 and GL3) from *Arabidopsis* can activate anthocyanin biosynthesis in a white-flowered *Matthiola incana* mutant. *Plant Mol. Biol.* 52 679–688. 10.1023/a:1024852021124 12956536

[B51] Riechmann J. Heard J. Martin G. Reuber L. Keddie J. Adam L. (2000). *Arabidopsis* transcription factors: genome-wide comparative analysis among eukaryotes. *Science* 290 2105–2110. 10.1126/science.290.5499.2105 11118137

[B52] Seo J. S. Joo J. Kim M. J. Kim Y. K. Nahm B. H. Song S. I. (2011). OsbHLH148, a basic helix-loop-helix protein, interacts with OsJAZ proteins in a jasmonate signaling pathway leading to drought tolerance in rice. *Plant J.* 65 907–921. 10.1111/j.1365-313X.2010.04477.x 21332845

[B53] Shannon P. Markiel A. Ozier O. Baliga N. S. Wang J. T. Ramage D. (2003). Cytoscape: a software environment for integrated models of biomolecular interaction networks. *Genome Res.* 13 2498–2504. 10.1101/gr.1239303 14597658 PMC403769

[B54] Shi L. Campbell G. Jones W. Campagne F. Wen Z. Walker S. (2010). The MicroArray Quality Control (MAQC)-II study of common practices for the development and validation of microarray-based predictive models. *Nat. Biotechnol.* 28 827–838. 10.1038/nbt.1665 20676074 PMC3315840

[B55] Shiu S. H. Bleecker A. B. (2003). Expansion of the receptor-like kinase/pelle gene family and receptor-like proteins in *Arabidopsis*. *Plant Physiol.* 132 530–543. 10.1104/pp.103.021964 12805585 PMC166995

[B56] Song C. Shan W. Kuang J. Chen J. Lu W. (2020). The basic helix-loop-helix transcription factor MabHLH7 positively regulates cell wall-modifying-related genes during banana fruit ripening. *Postharvest Biol. Technol.* 161:111068.

[B57] Song S. Qi T. Fan M. Zhang X. Gao H. Huang H. (2013). The bHLH subgroup IIId factors negatively regulate jasmonate-mediated plant defense and development. *PLoS Genet.* 9:e1003653 10.1371/journal.pgen.1003653 PMC3723532 23935516

[B58] Song X. M. Huang Z. N. Duan W. K. Ren J. Liu T. K. Li Y. (2014). Genome-wide analysis of the bHLH transcription factor family in Chinese cabbage (*Brassica rapa* ssp. pekinensis). *Mol. Genet. Genomics* 289 77–91. 10.1007/s00438-013-0791-3 24241166

[B59] Stover R. H. (1962). *Fusarium Wilt (Panama disease) of Banana and Other Musa Species.* Kew: Commonwealth Mycological Institute.

[B60] Sun H. Fan H. J. Ling H. Q. (2015). Genome-wide identification and characterization of the bHLH gene family in tomato. *BMC Genomics* 16:9 10.1186/s12864-014-1209-2 PMC4312455 25612924

[B61] Tamura K. Stecher G. Peterson D. Filipski A. Kumar S. (2013). MEGA6: molecular evolutionary genetics analysis version 6.0. *Mol. Biol. Evol.* 30 2725–2729. 10.1093/molbev/mst197 24132122 PMC3840312

[B62] Toledo-Ortiz G. Huq E. Quail P. H. (2003). The *Arabidopsis* basic/helix-loop-helix transcription factor family. *Plant Cell* 15 1749–1770. 12897250 10.1105/tpc.013839 PMC167167

[B63] Udyavar A. R. Hoeksema M. D. Clark J. E. Zou Y. Tang Z. Li Z. (2013). Co-expression network analysis identifies spleen tyrosine kinase (SYK) as a candidate oncogenic driver in a subset of small-cell lung cancer. *BMC Syst. Biol.* 7:S1 10.1186/1752-0509-7-S5-S1 PMC4029366 24564859

[B64] van Asten P. J. Fermont A. M. Taulya G. (2011). Drought is a major yield loss factor for rainfed East African highland banana. *Agric. Water Manag.* 98 541–552.

[B65] Visser M. Gordon T. Fourie G. Viljoen A. (2010). Characterisation of South African isolates of *Fusarium oxysporum* f. sp. cubense from *Cavendish bananas*. *S. Afr. J. Sci.* 106 01–06.

[B66] Wang L. Xiang L. Hong J. Xie Z. Li B. (2019). Genome-wide analysis of bHLH transcription factor family reveals their involvement in biotic and abiotic stress responses in wheat (*Triticum aestivum* L.). *3 Biotech* 9:236 10.1007/s13205-019-1742-4 PMC6536565 31139551

[B67] Wang Y. Jiang C. J. Li Y. Y. Wei C. L. Deng W. W. (2012). CsICE1 and CsCBF1: two transcription factors involved in cold responses in Camellia sinensis. *Plant Cell Rep.* 31 27–34. 10.1007/s00299-011-1136-5 21850593

[B68] Wang Z. Miao H. Liu J. Xu B. Yao X. Xu C. (2019). *Musa balbisiana* genome reveals subgenome evolution and functional divergence. *Nat. Plants* 5 810–821. 10.1038/s41477-019-0452-6 31308504 PMC6784884

[B69] Wang Z. Zhang J. Jia C. Liu J. Li Y. Yin X. (2012). *De novo* characterization of the banana root transcriptome and analysis of gene expression under *Fusarium oxysporum* f. sp. Cubense tropical race 4 infection. *BMC Genomics* 13:650 10.1186/1471-2164-13-650 PMC3534498 23170772

[B70] Xiao Y. Y. Kuang J. F. Qi X. N. Ye Y. J. Wu Z. X. Chen J. Y. (2018). A comprehensive investigation of starch degradation process and identification of a transcriptional activator Mab HLH 6 during banana fruit ripening. *Plant Biotechnol. J.* 16 151–164. 10.1111/pbi.12756 28500777 PMC5785343

[B71] Xu Y. Hu W. Liu J. Zhang J. Jia C. Miao H. (2014). A banana aquaporin gene, MaPIP1; 1, is involved in tolerance to drought and salt stresses. *BMC Plant Biol.* 14:59 10.1186/1471-2229-14-59 PMC4015420 24606771

[B72] Zhang B. Horvath S. (2005). A general framework for weighted gene co-expression network analysis. *Stat. Appl. Genet. Mol. Biol.* 4:17 10.2202/1544-6115.1128 16646834

[B73] Zhang C. Feng R. Ma R. Shen Z. Cai Z. Song Z. (2018). Genome-wide analysis of basic helix-loop-helix superfamily members in peach. *PLoS One* 13:e0195974 10.1371/journal.pone.0195974 PMC5901983 29659634

[B74] Zhang H. B. Bokowiec M. T. Rushton P. J. Han S. C. Timko M. P. (2012). Tobacco transcription factors NtMYC2a and NtMYC2b form nuclear complexes with the NtJAZ1 repressor and regulate multiple jasmonate-inducible steps in nicotine biosynthesis. *Mol. Plant* 5 73–84. 10.1093/mp/ssr056 21746701

[B75] Zhao M. L. Wang J. N. Shan W. Fan J. G. Kuang J. F. Wu K. Q. (2013). Induction of jasmonate signalling regulators MaMYC2s and their physical interactions with MaICE1 in methyl jasmonate-induced chilling tolerance in banana fruit. *Plant Cell Environ.* 36 30–51. 10.1111/j.1365-3040.2012.02551.x 22651394

[B76] Zheng Y. Jiao C. Sun H. Rosli H. G. Pombo M. A. Zhang P. (2016). iTAK: a program for genome-wide prediction and classification of plant transcription factors, transcriptional regulators, and protein kinases. *Mol. Plant* 9 1667–1670. 10.1016/j.molp.2016.09.014 27717919

[B77] Zhou J. Li F. Wang J. L. Ma Y. Chong K. Xu Y. Y. (2009). Basic helix-loop-helix transcription factor from wild rice (OrbHLH2) improves tolerance to salt-and osmotic stress in *Arabidopsis*. *J. Plant Physiol.* 166 1296–1306. 10.1016/j.jplph.2009.02.007 19324458

